# Optimizing Livers for Transplantation Using Machine Perfusion versus Cold Storage in Large Animal Studies and Human Studies: A Systematic Review and Meta-Analysis

**DOI:** 10.1155/2018/9180757

**Published:** 2018-09-05

**Authors:** Xinan Jiang, Lei Feng, Mingxin Pan, Yi Gao

**Affiliations:** ^1^Department of Hepatobiliary Surgery II, Guangdong Provincial Research Center for Artificial Organ and Tissue Engineering, Guangzhou Clinical Research and Transformation Center for Artificial Liver, Institute of Regenerative Medicine, Zhujiang Hospital, Southern Medical University, Guangzhou 510280, Guangdong Province, China; ^2^Department of Urology Surgery, The Affiliated Hospital of Guizhou Medical University, Guiyang 550004, Guizhou Province, China

## Abstract

**Background:**

Liver allograft preservation frequently involves static cold storage (CS) and machine perfusion (MP). With its increasing popularity, we investigated whether MP was superior to CS in terms of beneficial outcomes.

**Methods:**

Human studies and large animal studies that optimized livers for transplantation using MP versus CS were assessed (PubMed/Medline/EMBASE). Meta-analyses were conducted for comparisons. Study quality was assessed according to the Newcastle-Ottawa quality assessment scale and SYRCLE's risk of bias tool.

**Results:**

Nineteen studies were included. Among the large animal studies, lower levels of lactate dehydrogenase (SMD -3.16, 95% CI -5.14 to -1.18), alanine transferase (SMD -2.46, 95% CI -4.03 to -0.90), and hyaluronic acid (SMD -2.48, 95% CI -4.21 to -0.74) were observed in SNMP-preserved compared to CS-preserved livers. NMP-preserved livers showing lower level of hyaluronic acid (SMD -3.97, 95% CI -5.46 to -2.47) compared to CS-preserved livers. Biliary complications (RR 0.45, 95% CI 0.28 to 0.73) and early graft dysfunction (RR 0.56, 95% CI 0.34 to 0.92) also significantly reduced with HMP preservation in human studies. No evidence of publication bias was found.

**Conclusions:**

MP preservation could improve short-term outcomes after transplantation compared to CS preservation. Additional randomized controlled trials (RCTs) are needed to develop clinical applications of MP preservation.

## 1. Introduction

The optimally effective treatment for end-stage liver disease remains liver transplantation [1]. With the development of perioperative treatment methods, surgical techniques, and posttransplant immunosuppression regimens, the survival rate following liver transplantation has improved, with the incidence of complications having significantly decreased [2]. However, an imbalanced supply and demand situation for suitable organs arose worldwide, which limited widespread use of this technique. This imbalance promoted the development of different strategies to expand donor pools and optimize livers for liver transplantation.

Cold static storage (CS) has become the primary organ preservation strategy since the development of specialized preservation solutions, especially the University of Wisconsin (UW) solution [1]. However, there are several limitations to CS, including cold ischemia-related organ damage, difficulty assessing donor organ function and viability before the transplant and limited opportunity to repair organ function [2, 3].

Machine perfusion (MP) comprises normothermic (NMP, 35–38°C), hypothermic (HMP, 4–10°C), and subnormothermic (SNMP, 20–30°C) methods according to perfusion temperature [1]. However, with specialized preservation solutions, MP has gradually been replaced by CS because MP is a complex and expensive process. Recently, MP has regained popularity owing to the changing donor profiles and updated perfusion solutions and surgical technologies [4]. However, the application of MP is still not widespread, with conflicting evidence regarding its utility. Additionally, the preservation mechanism involved is still poorly understood. Many studies used small animals [5–7] or simulated reperfusion* in vitro* rather than* in vivo *transplants [8–10]. Thus, the performance of the transplanted liver in actual conditions of human liver transplantation remains unclear. Whether MP is superior to CS is still unknown.

Accordingly, we conducted a meta-analysis of large animal studies and human studies of liver transplantation after preservation to determine whether MP provided more beneficial outcomes than CS. We also explored the underlying mechanisms of action of MP preservation.

## 2. Materials and Methods

The meta-analyses in this study were performed according to the guidelines of the Preferred Reporting Items for Systematic Reviews and Meta-Analyses (PRISMA) statement.

### 2.1. Literature Search and Eligibility Criteria

The PubMed, Medline, and EMBASE databases were searched using the MeSH terms or key words including “liver or hepatic” and “machine perfusion” and dates between January 1980 and April 2018. In order to include all eligible studies, we also performed a manual literature search using any potential articles' bibliographies and reference lists from other reviews.

Inclusion criteria were as follows: (1) all studies (human and animal) that included different types of MP and CS preservations were eligible for this analysis, (2) both English and non-English articles were considered, (3) the optimized livers must have been transplanted after MP or CS preservation, (4) only published works were included, and (5) species were limited to humans and pigs.

Exclusion criteria were as follows: (1) human studies with insufficient data, (2) studies without a control group (CS preservation group), (3) studies which only used simulated reperfusion* in vitro* after MP or CS preservation, and (4) all studies conducted prior to 1980.

### 2.2. Data Extraction

Two independent reviewers (Feng L, Jiang XN) screened the titles and abstracts of all citations. The full text articles were retrieved for comprehensive review and then were rescreened. A third reviewer (Gao Y) was consulted if necessary for any disagreements between the two independent reviewers.

Human data were analyzed for extraction of the following: first author and date of publication; number of patients in the MP and CS groups; stratification of MP by HMP, NMP, and SNMP status; MP characteristics, including oxygenation, preservation temperature, and perfusion pressure; preservation solution(s) used in the CS and MP groups; and outcomes. Quantitative data were extracted to determine the incidence of early graft dysfunction (EAD) and primary nonfunction (PNF), international normalized ratio (INR), peak aspartate aminotransferase (AST) level, hospital stay, intensive care unit (ICU) stay, and graft survival in the human studies.

The early graft dysfunction (EAD) was defined as when one or more of the following variables were present: (1) bilirubin ≧ 10 mg/dL on postoperative day 7; (2) INR ≧ 1.6 on postoperative day 7; (3) aminotransferase level (ALT or AST) >2000 IU/mL within the first 7 postoperative days [11, 12]. The primary nonfunction (PNF) was defined as progressive increases in serum transaminases within 48h after OLT, uncorrectable coagulopathy, metabolic acidosis, and the hepatorenal syndrome [13, 14].

The study parameters collected for large animal data included first author and date of publication; number of patients in the MP and CS groups; stratification of MP by HMP, NMP, and SNMP status; MP characteristics, including oxygenation, preservation temperature, and perfusion pressure; preservation solution(s) used in the CS and MP groups; and outcomes. Study outcomes included liver function parameters (levels of peak alanine transferase (ALT), peak AST, and peak lactate dehydrogenase (LDH)), sinusoidal endothelial injury parameter (HA), and animal survival.

The survival time in animal studies was defined according to each study's protocol. Data presented in medians and ranges were converted into means and standard deviations (SD) using a method described previously [15]. If different intervention groups (HMP, NMP, or SNMP) were compared to one control group (CS), or one intervention group was compared to a different CS, we treated them as different studies.

### 2.3. Quality Assessment

A quality assessment of the clinical studies included in the meta-analysis was performed using the Newcastle-Ottawa quality assessment scale [16]. Animal studies are generally different from human studies. Therefore, the Systematic Review Centre for Laboratory Animal Experimentation (SYRCLE) risk of bias tool for animal studies was utilized to assess the quality of animal data included in meta-analysis [17].

### 2.4. Statistical Analysis

Meta-analyses were performed for the above comparator groups using STATA software version 12.0 (STATACorp LLC, College Station, TX, USA). The I^2^ statistic and P value of the Q test were used to analyze study heterogeneity, with I^2^>50% or P ≦ 0.1 and I^2^ ≧ 25% indicating high levels of heterogeneity. In these cases, a random-effects model was used; otherwise, a fixed-effects model was employed. The results were calculated as risk ratios (RR) with 95% confidence intervals (CI) for dichotomous data. Measurement data results were calculated as standardized mean differences (SMD) with a 95% CI. We conducted subgroup analyses based on WIT and donor type. Sensitivity analysis was also performed to confirm whether the same trend was observed with the remaining trials after removed the included studies one-by-one. Publication bias was examined in funnel plots by performing Begg's test. A P value <0.05 indicated statistical significance.

## 3. Results

### 3.1. Summary of Animal and Human Study Characteristics

**Figure 1:**
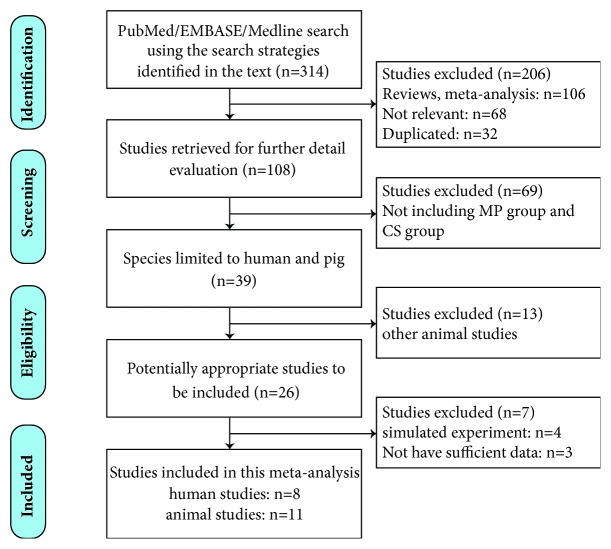
Study selection flow diagram. MP: machine perfusion; CS: cold static storage.

In this systematic review, we analyzed both human and animal studies. According to the abovementioned strategies, we initially identified 314 articles in PubMed/EMBASE/Medline. After removing reviews, meta-analyses, duplicates, and irrelevant articles from the searched articles, a total of 108 articles were left for further detailed evaluation. After evaluating the article abstracts, 69 studies were removed because there were no CS groups. Thirteen articles were excluded after we limited species to humans and pigs. A total of 26 potential studies were read in full text. Seven of these studies were excluded after reading the full text; among these, 4 studies[8–10, 18] used simulated reperfusion* in vitro* instead of liver transplantation and 3 studies [19–21] had insufficient data. Finally, 19 studies (8 human studies and 11 animal studies) were included in this meta-analysis, as shown in the study selection flow diagram (Figure 1). Among these studies, three of the animal studies [22–24] had different intervention groups compared to the control group and one of the human studies [25] had an intervention group compared to a different control group. Thus, we treated these as different studies when extracting data.

Baseline study characteristics are presented in Table 1. The study quality evaluation according to SYRCLE's risk of bias tool for animal studies and the Newcastle-Ottawa quality assessment scale for human studies is outlined in Tables 2 and 3, respectively.

### 3.2. Results from the Animal Studies

#### 3.2.1. Effect of MP on Liver Function

**Figure 2:**
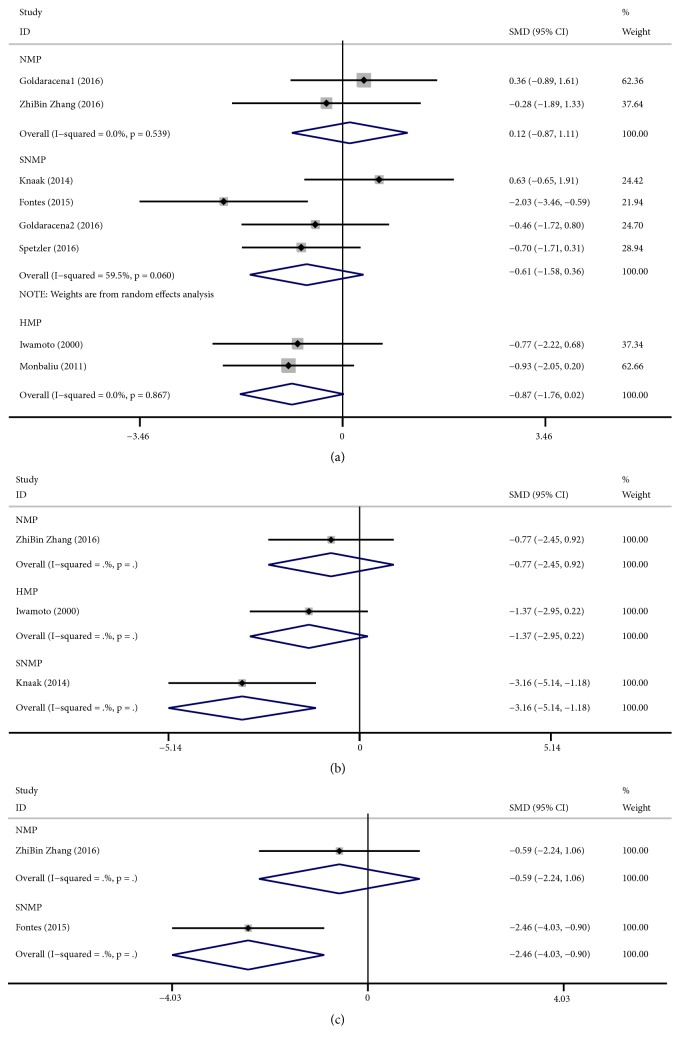
Forest plots comparing peak AST, Peak LDH, and ALT for all studies comparing different MP to CS in animal studies.** (a)** Peak AST. NMP (SMD: 0.12, 95% CI:-0.87 to 1.11) increased the release of AST, while SNMP (SMD: -0.61, 95% CI:-1.58 to 0.36) and HMP (SMD: -0.87, 95% CI:-1.76 to 0.02) reduced the release of AST;** (b)** peak LDH. NMP (SMD: -0.77, 95% CI: -2.45 to 0.92), HMP (SMD:-1.37, 95% CI: -2.95 to 0.22), and SNMP (SMD: -3.16, 95% CI: -5.14 to -1.18) reduced the release of LDH;** (c)** ALT. NMP (SMD:-0.59, 95% CI: -2.24 to 1.06) and SNMP (SMD:-2.46, 95% CI: -4.03 to -0.90) reduced the release of ALT. AST: aspartate aminotransferase; LDH: lactate dehydrogenase; ALT: alanine aminotransferase; MP: machine perfusion; HMP: hypothermic machine perfusion; NMP: normothermic machine perfusion; SNMP: subnormothermic machine perfusion; CS: cold static storage; SMD: standardized mean differences.

Postpreservation liver function in the animal studies was assessed using the parameters of peak AST, LDH and ALT. There were 7 studies that reported a release of peak AST after liver transplantation. After combining the results of the studies that measured AST levels, the meta-analysis showed that the SMD of peak AST was lower with HMP (fixed-effects analysis: SMD -0.87, 95% CI -1.76 to 0.02),SNMP (fixed-effects analysis: SMD -0.61, 95% CI -1.58 to 0.36) than with CS, but the difference had no statistically significant (HMP: P=0.055; SNMP: P=0.221). Meanwhile, NMP (random-effects analysis: SMD 0.12, 95% CI -0.87 to 1.11) did not show any benefit than with CS. There was significant heterogeneity in SNMP (I^2^ = 59.5%, P = 0.060) (Figure 2(a)). Subgroup analysis of AST in SNMP showed that according to the WIT and donor type, most of the 95% CI between the subgroups was overlapped, meaning that there was no significant difference between the subgroups (Supplementary 1).

There were only three studies that reported the release of LDH. The meta-analysis showed that the SMD of LDH was lower with NMP (fixed-effects analysis: SMD -0.77, 95% CI -2.45 to 0.92), HMP (fixed-effects analysis: SMD -1.37, 95% CI -2.95 to 0.22), and SNMP (fixed-effects analysis: SMD -3.16, 95% CI -5.14 to -1.18) than with CS. However, only the difference in SNMP showed statistically significance (P=0.002) (Figure 2(b)).

Furthermore, we also evaluated the release of ALT, and there were only two studies that reported the release of ALT after transplantation. The meta-analysis showed that the SMD of peak ALT was lower with NMP (fixed-effects analysis: SMD -0.59, 95% CI -2.24 to 1.06) and SNMP (fixed-effects analysis: SMD -2.46, 95% CI -4.03 to -0.90) than with CS. However, only the difference in SNMP was statistically significant (P=0.002) (Figure 2(c)).

#### 3.2.2. Effect of MP on Sinusoidal Endothelial Cells and Animal Survival

**Figure 3:**
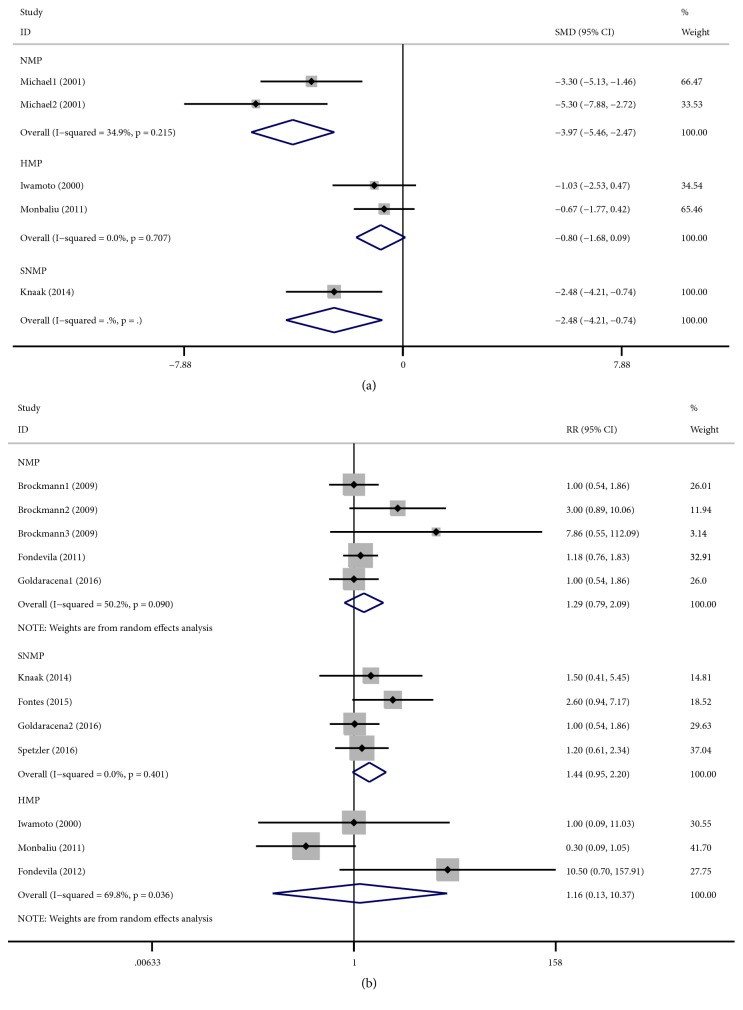
Forest plots comparing HA and animal survival for all studies comparing different MP to CS in animal studies.** (a)** HA. NMP (SMD: -3.97, 95% CI:-5.46 to -2.47), HMP (SMD: -0.80, 95% CI:-1.68 to 0.09), and SNMP (SMD: -2.48, 95% CI:-4.21 to -0.74) reduced the release of HA;** (b)** animal survival. NMP (RR: 1.29, 95% CI: 0.79 to 2.09), HMP (SMD: 1.16, 95% CI: 0.13 to 10.37), and SNMP (SMD: 1.44, 95% CI: 0.95 to 2.20) improved the survival. HA: hyaluronic acid; MP: machine perfusion; HMP: hypothermic machine perfusion; NMP: normothermic machine perfusion; SNMP: subnormothermic machine perfusion; CS: cold static storage; SMD: standardized mean differences; CI: confidence interval; RR: relative risk.

The evaluation of sinusoidal endothelial cell damage was presented using hyaluronic acid (HA). There were four studies that reported the levels of HA. The livers of animals in studies with NMP (fixed-effects analysis: SMD -3.97, 95% CI -5.46 to -2.47; P< 0.001), HMP (fixed-effects analysis: SMD -0.80, 95% CI -1.68 to -0.09; P=0.077), and SNMP (fixed-effects analysis: SMD -2.48, 95% CI -4.21 to -0.74; P=0.005) preservation presented with lower HA levels than that of the CS group (Figure 3(a)).

Reports of survival in animal studies are not documented to present actual survival per se but are more of a reflection of the maintenance of liver function because the great majority of deaths occurred by euthanasia after features indicating liver failure appeared. Meta-analysis showed that the RR of animal survival was higher with NMP (random-effects analysis: RR 1.29, 95% CI 0.79 to 2.09; P=0.314), HMP (random-effects analysis: RR 1.16, 95% CI 0.13 to 10.37; P=0.893), and SNMP (fixed-effects analysis: RR 1.44, 95% CI 0.95 to 2.20; P=0.086) than with CS. However, the difference had no statistically significant. There was significant heterogeneity in NMP (I^2^ = 50.2%, P < 0.001) and HMP (I^2^ = 69.8%, P = 0.036) (Figure 3(b)). Subgroup analysis showed no significant difference between the subgroups (Supplementary 2 and 3).

### 3.3. Results from Human Studies

#### 3.3.1. Effect of MP on Biliary Complications, EAD, PNF, and Graft Survival

**Figure 4:**
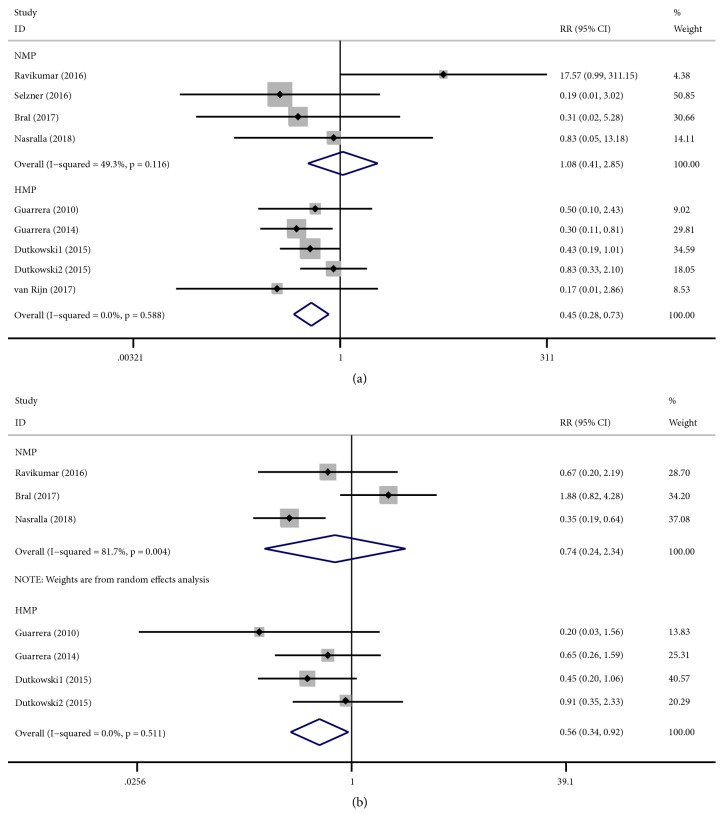
Forest plots comparing biliary complications and EAD for all studies comparing different MP to CS in human studies.** (a)** Biliary complications. NMP cannot reduce the biliary complications (RR: 1.08, 95% CI: 0.41 to 2.85); HMP reduced the biliary complications (RR: 0.45, 95% CI: 0.28 to 0.73);** (b)** EAD. NMP reduced the EAD (RR: 0.74, 95% CI: 0.24 to 2.34) and HMP reduced the EAD (RR: 0.56, 95% CI: 0.34 to 0.92). MP: machine perfusion; HMP: hypothermic machine perfusion; NMP: normothermic machine perfusion; CS: cold static storage; SMD: standardized mean differences; RR: relative risk; CI: confidence interval; EAD: early allograft dysfunction.

All human studies included in this meta-analysis reported the occurrence of biliary complications. Biliary complication rates were significantly lower in human studies utilizing HMP (fixed-effects analysis: RR 0.45, 95% CI 0.28 to 0.73; P=0.001) compared to those of CS. There was no significant heterogeneity (I^2^ = 0.0%; Cochran's Q =2.82, P = 0.558). However, NMP (fixed-effects analysis: RR 1.08, 95% CI 0.41 to 2.85; P=0.878) did not show any benefit than with CS. There was no significant heterogeneity (I^2^ = 49.3%; Cochran's Q =5.92, P = 0.116) (Figure 4(a)).

There were five studies reporting early allograft dysfunction (EAD). EAD was significantly lower in human studies utilizing NMP (random-effects analysis: RR 0.74, 95% CI 0.24 to 2.34; P=0.614) and HMP (fixed-effects analysis: RR 0.56, 95% CI 0.34 to 0.92; P=0.021) compared to those of CS. However, only the difference in HMP had statistical significance (P=0.021). There was significant heterogeneity in NMP (I^2^ = 81.7%; Cochran's Q = 10.90, P = 0.004) (Figure 4(b)). Subgroup analysis showed no significant difference between the subgroups (Supplementary 4).

**Figure 5:**
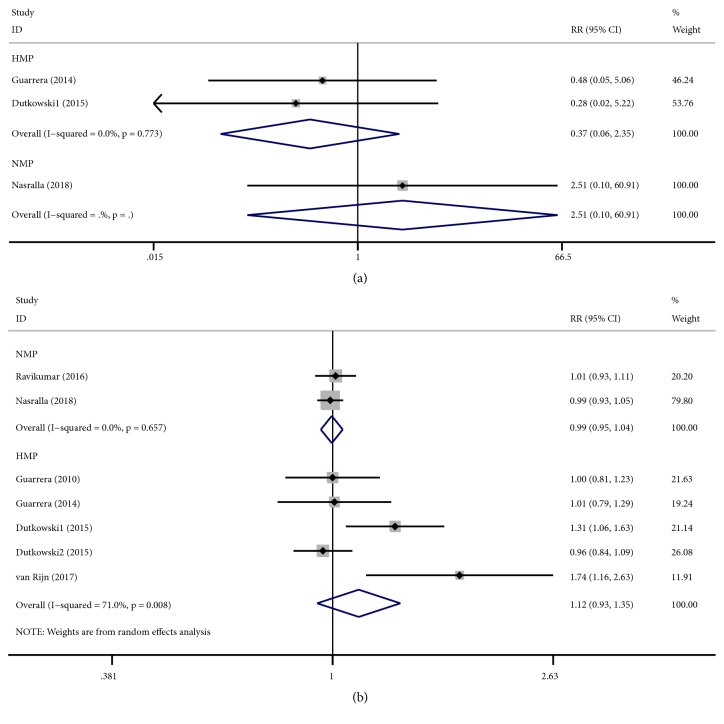
Forest plots comparing PNF and graft survival for all studies comparing different MP to CS in human studies.** (a)** PNF. HMP reduced the PNF (RR: 0.37, 95% CI: 0.06 to 2.35), while NMP increased the PNF (RR: 2.51, 95% CI: 0.10 to 60.91);** (b)** graft survival. NMP cannot improve the graft survival (RR: 0.99, 95% CI: 0.95 to 1.04), while HMP improved the graft survival (RR: 1.12, 95% CI: 0.93 to 1.35). MP: machine perfusion; HMP: hypothermic machine perfusion; NMP: normothermic machine perfusion; CS: cold static storage; PNF: primary nonfunction; RR: relative risk; CI: confidence interval.

There were only three studies that reported PNF. PNF were lower in human studies utilizing NMP (fixed-effects analysis: RR 2.51, 95% CI 0.10 to 60.91; P=0.572) and HMP (fixed-effects analysis: RR 0.37, 95% CI 0.06 to 2.35; P=0.294) compared to those of CS. However, both of them had no statistical significance (Figure 5(a)).

There were six studies that reported graft survival. Graft survival was lower in human studies utilizing NMP (fixed-effects analysis: RR 0.99, 95% CI 0.95 to 1.04; P=0.821) compared to those of CS, while it was in HMP (random-effects analysis: RR 1.12, 95% CI 0.93 to 1.35; P=0.218) higher when compared to those of CS. However, both of them had no statistical significance. There was significant heterogeneity in HMP (I^2^ = 71.0%; Cochran's Q = 13.80, P = 0.008) (Figure 5(b)). Subgroup analysis showed no significant difference between the subgroups (Supplementary 5)

#### 3.3.2. Effect of MP on Hospital Stay, ICU Stay, Peak AST, and INR

All human studies included in this meta-analysis reported the lengths of hospital stay. Lengths of hospital stays were decreased in human studies utilizing HMP (random-effects analysis: SMD -0.088, 95% CI -0.662 to 0.485; P=0.762) when compared to CS. However, the difference showed no statistical significance (P=0.751), while in NMP (random-effects analysis: SMD 0.338, 95% CI -0.286 to 0.963; P=0.288) it was higher when compared to those of CS (Supplementary 6).

Five studies reported ICU stays. ICU stays were significantly longer in human studies utilizing NMP (random-effects analysis: SMD 0.563, 95% CI -0.330 to 1.457; P=0.217) and HMP (fixed-effects analysis: SMD 0.307, 95% CI -0.004 to 0.619; P=0.053) when compared with CS. However, both of them had no statistical significance (Supplementary 6).

Five studies reported the peak AST. Peak AST was higher in human studies utilizing NMP (random-effects analysis: SMD -4.616, 95% CI -10.364 to 1.133; P=0.116) and HMP (random-effects analysis: SMD -1.235, 95% CI -3.333 to 0.863; P=0.249) when compared with CS. However, both of them had no statistical significance (Supplementary 6).

Similarly, as a marker of liver function, the meta-analysis of INR showed no significant difference in NMP (SMD -0.083, 95% CI -0.133 to 0.299; P=0.453) and HMP (SMD -0.594, 95% CI -2.683 to 1.495; P=0.577) when compared to CS. However, both of them had no statistical significance (Supplementary 6).

### 3.4. Publication Bias in All Studies

We also assessed the publication bias regarding the effect of different MP preservation on all the selected studies using a Begg's test. No evidence of publication bias was found in the selected indicators used to evaluate different MP preservation in animal and human studies except graft survival in HMP (Supplementary 7).

## 4. Discussion

This meta-analysis provides a comprehensive understanding of the current published literature regarding different MPs preservation of liver grafts versus cold storage prior to transplantation in large animal studies and clinical studies. Large animal study data showed that MP preservation significantly decreased ALT, LDH, and HA values compared to CS preservation, which is in accordance with previous meta-analyses [2, 41]. However, previous meta-analyses also included small animal studies [2] or studies that only simulated reperfusion* in vitro* rather than with transplants* in vivo* [41]. These methods cannot truly reflect the real conditions that occur after clinical transplantation. The clinical study data in our meta-analysis showed that HMP preservation significantly decreased biliary complications and EAD compared to CS preservation.

Currently, CS preservation is the most commonly used pretransplantation strategy because of its simplicity and low cost. However, with donor organ demand exceeding supply, MP preservation has been an attractive option that provides all of the native liver functions including the opportunity for waste product metabolism and excretion as well as the provision of oxygen and nutrients and revival of liver metabolic function following warm ischemia and cold storage damage [4]. Furthermore, it could allow for measurement of donor organ viability* in vitro* prior to transplantation [4].

Perfusion temperature plays an important role in MP preservation. NMP maintains the liver* ex vivo* on a circuit by providing oxygen and nutrition at 35-38°C, a condition that more closely approximates physiological conditions [42]. However, the liver in NMP has greater oxygen and nutrition needs because of the rapid metabolic function. Therefore, the perfusion solution must have strong oxygen carrying capacity, and the most commonly used is whole blood. Unfortunately, shortage of clinical blood may limit NMP applications, although some research teams have tried to develop new perfusion solutions [24, 31]. SNMP (20-30°C) is developed from NMP without oxygen carriers (such as blood), and therefore it may overcome the limitations associated with NMP in a widespread application. Although some studies manifested feasibility applications of SNMP [43], more studies are needed to verify this. HMP (4–10°C) is closer to CS, except for continuous perfusion and metabolic product excretion. This process triggers endothelial protection via upregulation of shear stress-sensitive protective genes or by triggering a unique decrease in damage-associated molecular patterns (DAMPs) during early reperfusion [44–46].

Liver function in donor organs is related to the recipient's prognosis after liver transplantation. Many enzymes are released into the blood when hepatocytes and mitochondrial membranes are damaged [41]. ALT, AST, and LDH are the most frequently used markers to assess liver function. If the liver experiences serious damage, the serum ALT, AST, and LDH levels will increase. Our meta-analysis results showed that MP preservation, especially SNMP, significantly decreased serum ALT and LDH levels when compared to CS preservation. Thus, MP could protect the liver and its function from cold storage damage, which prolongs the preservation time.

HA is the most commonly used marker of sinusoidal endothelial cell (SEC) function. When flow cessation results in a significant reduction of endothelial vasoprotective pathways leading to cell activation and apoptosis [45], the SECs are severely destroyed and the serum HA level will be significantly increased. Our meta-analysis results showed that SNMP and NMP preservation significantly decreased serum HA levels when compared to CS preservation in animal studies. Therefore, MP preservation can protect SEC from ischemic injury. However, there have been no clinical studies that have measured the level of serum HA.

With the development of liver transplantation, more and more patients are faced with a shortage of suitable organs. Therefore, it is very important to expand the donor pools. One strategy is to use extended criteria donor (ECD) grafts, such as donation after circulatory death (DCD), steatotic, or grafts from elderly persons [1]. However, these marginal organs have increased susceptibility to ischemia reperfusion injury (IRI), leading to high risk of PNF, EAD, and biliary complications after transplantation. Our meta-analysis results showed that HMP preservation significantly decreased EAD and biliary complications compared to CS preservation in clinical studies. This indicates that MP preservation can optimize the usable livers for transplantation and, eventually, this could expand the donor pools.

We also assessed animal survival in animal studies and graft survival in clinical studies. However, our results showed no significant differences between different MP and CS preservation. This result is in accordance with previous meta-analyses [41]. Because the included study was small, the conclusion may have been incorrect. Therefore, the effect of different MP on posttransplant graft survival rates and long-term efficacy still needs further research.

ICU and hospital stays affect costs after liver transplantation in clinical trials. In the present meta-analysis, we also assess ICU and hospital stays. However, our results showed no significant differences between different MP preservation and CS preservation with regard to postoperative lengths of hospital stay and ICU stay. This result is in accordance with a recent published RCT study [40]. Our included studies used medians and ranges to present the ICU and hospital stay data; however, we converted these data into means and SDs using an approximation method, which may have led to incorrect results.

In our meta-analysis, there was a high degree of heterogeneity in the analyses of peak AST (SNMP) and animal survival (NMP and HMP) in the animal studies and in graft survival rate (HMP), EAD (NMP), hospital stay (NMP), ICU stay (NMP), peak AST (NMP and HMP), and INR (HMP) in the clinical studies. Subgroup analyses were performed according to WIT and donor type. These subgroups' analysis showed that heterogeneity could not be eliminated by grouping, which made us think that the WIT and donor type were not the main sources of heterogeneity.

 However, a variety of solutions used were different to the CS control, which may impact the overall effect of MP. Otherwise, the perfusion conditions, such as perfusion pressure and whether oxygenate, and operation methods used were different. While in human studies, there was only one RCT study included in this meta-analysis, different study designs of non-RCT studies (poor blinding and allocation concealment) arose high heterogeneity. We think that all of these were the main source of heterogeneity.

In order to reduce type I error, a random-effects model was used to account for any study heterogeneity. Furthermore, the sensitivity analysis, which is the included studies in different groups (NMP, SNMP, and HMP) removed one-by-one, showed that the final results did not change significantly. Therefore, we think that the results of our meta-analysis are reliable.

Our meta-analysis has some limitations. First, we used an approximation method as described previously, to deal with data presented as medians and ranges, which may have led to incorrect results. Second, a relatively small number of studies with small sample sizes were included in the present meta-analysis. Third, we included animal studies which were mainly focused on the short-term effects of the model. Finally, out of the clinical studies included in our analysis only one is RCT study. All of the abovementioned factors may have affected the final results. Additional high quality studies are needed to confirm our results.

## 5. Conclusions

In conclusion, MP preservation could improve short-term outcomes after liver transplantation compared to CS preservation. More studies are needed to develop the clinical application of MP preservation. Our findings may provide more data to aid in choosing suitable organ preservation strategy before transplantation or transportation for use in clinical practice.

## Figures and Tables

**Table 1:** Characteristics of the large animal studies and human studies included in this meta-analysis.

References	Species	Donor type	WIT	MP type	Numbers (n)		MP characteristics	Preservation solution		Outcomes
					MP	CS		MP	CS	
Iwamoto [26] (2000)	pig	DCD	NR	HMP	4	4	8°C, 2h, PV:7-8mmHg	UW-G	UW	Survival, AST, HA,LDH
Michael [22] (2001)	pig	DCD	0/1h	NMP	6/6	6/6	37°C, 4h, LA:150ml/min, PV:250ml/min	Blood	UW	Survival, PNF, HA,INR
Brockmann [23] (2009)	pig	HBD/ DCD	0/0/40 min	NMP	5/7/6	5/7/4	5/20h, LA:240ml/min, PV: 7.22mmHg	Blood	UW	Survival, AST, ALT, HA
Fondevila [27] (2011)	pig	DCD	25min	NMP	6	6	35.3-37.5°C, 4h, LA: 40-60mm Hg, PV:8mmHg	Blood	UW	Survival, AST, bilirubin
Monbaliu [28] (2011)	pig	DCD	NR	HMP	8	6	4°C, 4h, LA:30mmHg, PV:7mmHg, nonoxygenated	UW	UW	Survival, PNF, AST, HA, TNF-*α*
Fondevila [29] (2012)	pig	DCD	26min	HMP	5	6	4°C, 4h, LA:20-30mmHg, PV:4mmHg	UW	UW	Survival, AST, HA, bilirubin, bile salts
Knaak[30] (2014)	pig	DCD	45min	SNMP	5	5	33°C, 3h, LA:60-80mm Hg,PV:4-8mmHg	Steen+ erythrocyte	UW	Survival, AST, HA, LDH, CD31,INR and factor V
Fontes [31] (2015)	pig	DCD	35min	SNMP	6	6	21°C, 7.5h, LA:18±2 mmHg, PV:3.5±0.5mmHg	HBOC solution	UW	Survival, AST, ALT, HA, LDH, Bile production
Spetzler [32] (2016)	pig	DCD	NR	SNMP	8	8	33°C, 3h, LA:50-60mmHg, PV:2-4mmHg	UW	UW	Survival, AST, ALT, bilirubin
Zhibin Zhang [33] (2016)	pig	DCD	0min	NMP	6	6	37°C, LA:85-100mmHg, PV:8-10mmHg	Blood	UW	AST, ALT, LDH
Goldaracena [24] (2016)	pig	HBD	NR	NMP/ SNMP	5/5	5/5	33-37°C,4h, LA:60mmHg, PV:2-4mmHg	Steen+ erythrocyte	Steen solution	Survival, AST, HA, CD31, IL-6, TNF-*α*
Guarrera [34] (2010)	human	DCD	NR	HMP	20	20	4-6°C, 3-7h, Flow rates were0.667 ml/g liver/min	vasosol solution	UW	Graft survival, PNF,EAD,biliary complications, INR, AST, hospital stay
Guarrera [35] (2015)	human	ECD	NR	HMP	31	30	4-6°C, 3-7h, Flow rates were0.667 ml/g liver/min	vasosol solution	UW	Graft survival, PNF, EAD,biliary complications, INR, AST, hospital stay
Dutkowski [25] (2015)	human	DCD/ DBD	36min	HMP	25/25	50/50	10°C, 1.5-2.5h, PV: 120–180 ml/min	UW	UW	Graft survival, PNF,EAD,biliary complications, INR, AST, hospital stay, ICU stay
Ravikumar [36] (2016)	human	DCD/ DBD	21min	NMP	20	40	37°C, 4h, LA: 60-75mmHg, PV:-1-2mmHg	Blood	UW	Graft survival, PNF, EAD, INR, hospital stay, AST, ICU stay
Selzner [37] (2016)	human	HBD/ DCD	49min	NMP	10	30	37°C, 4h, LA:200-400ml/min, PV:1200-1300ml/min	Steen solution	HTK/UW	Graft survival, INR, AST, hospital stay, ICU stay
Bral [38] (2017)	human	DCD/DBD	0-26min	NMP	9	27	37°C, 3.3-22.5h, LA and PV not reported	Blood	HTK/UW	Graft survival, PNF, EAD,biliary complications, INR, AST, hospital stay, ICU stay
van Rijn [39] (2017)	human	DCD	15min	HMP	10	20	10°C, 2h, LA: 20-30mmHg, PV: 5mmHg	UWMPS	UW	Graft survival, hospital stay, ICU stay, biliary complications
Nasralla [40] (2018)	human	DCD/ DBD	21min	NMP	121	101	37°C, 24h, LA: 200-400ml/ min, PV: 1000-1200ml/min	Blood	UW	Graft survival, hospital stay, ICU stay, AST, EAD, biliary complications,PNF

DCD: donation after circulatory death; HBD: heart beating donor; DBD: donation after brain death; WIT: warm ischemia time; MP: machine perfusion; CS: cold (static) storage; HMP: hypothermic machine perfusion; NMP: normothermic machine perfusion; SNMP: subnormothermic machine perfusion; LA: hepatic artery; PV: portal vein; UW: University of Wisconsin; UW-G: University of Wisconsin gluconate solution; HTK: histidine tryptophan ketoglutarate; HBOC: hemoglobin-based oxygen carrier; PNF: primary nonfunction; EAD: early allograft dysfunction; INR: international normalized ratio; HA: hyaluronic acid; ICU: intensive Care Unit; AST: aspartate amino-transferase; ALT: alanine transaminase; LDH: lactate dehydrogenase; CD31: cluster of differentiation 31; IL-6: interleukin-6; TNF-*α*: tumour necrosis factor *α*; NR: not report

**Table 2:** Quality of animal studies included in this analysis using SYRCLE's risk of bias tool for animal studies.

**Author( year)**	**(1)**	**(2)**	**(3)**	**(4)**	**(5)**	**(6)**	**(7)**	**(8)**	**(9)**	**(10)**
Iwamoto (2000)	Unclear	Unclear	Unclear	Unclear	Unclear	Yes	Unclear	Yes	No	Unclear
Michael (2001)	Yes	Yes	Unclear	Unclear	Unclear	Yes	Unclear	Yes	Yes	Unclear
Brockmann (2009)	Yes	Yes	Unclear	Unclear	Unclear	Yes	Unclear	Yes	Yes	Unclear
Fondevila (2011)	Yes	Yes	Unclear	Unclear	Unclear	Yes	Unclear	Yes	Yes	Unclear
Monbaliu (2011)	Unclear	Yes	Unclear	Unclear	Unclear	Yes	Unclear	Yes	Yes	Unclear
Fondevila (2012)	Yes	Yes	Unclear	Unclear	Unclear	Yes	Unclear	Unclear	No	Unclear
Knaak (2014)	Yes	Yes	Unclear	Unclear	Unclear	Yes	Unclear	No	No	Unclear
Fontes (2015)	Yes	Yes	Unclear	Unclear	Unclear	Yes	Unclear	Yes	No	Unclear
Spetzler (2016)	Yes	Yes	Unclear	Unclear	Unclear	Yes	Unclear	No	Yes	Unclear
Zhibin Zhang (2016)	Yes	Yes	Unclear	Unclear	Unclear	Yes	Unclear	No	Yes	Unclear
Goldaracena (2016)	Yes	Yes	Unclear	Unclear	Unclear	Yes	Unclear	No	Yes	Unclear

Study quality items are (1) adequate sequence generation; (2) similar baseline characteristics for study groups; (3) allocation concealment present; (4) random housing utilised; (5) blinding of investigators; (6) all animals selected for outcome assessment; (7) blinding of outcome assessor(s); (8) incomplete outcome data addressed; (9) outcome reporting not selective; (10) other sources of bias present.

**Table 3:** Quality for cohort studies included in the human meta-analysis using the Newcastle-Ottawa Quality Assessment Scale.

**Studies**	**Selection**				**Comparability**	**Assessment of Outcome**			**Scores**
**Author( year)**	**Representativeness of exposed cohort**	**Selection of Non- exposed cohort**	**Ascertainment of exposure**	**Demonstration that outcome of interest was not present at start of study**	**Comparability of cohorts in design and analysis**	**Assessment of outcome **	**Was follow-up long enough for outcomes to occur?**	**Adequacy of follow up**	
Guarrera (2010)	**☆**		**☆**	**☆**		**☆**	**☆**	**☆**	**6**
Guarrera (2014)	**☆**		**☆**	**☆**		**☆**	**☆**	**☆**	**6**
Dutkowski (2015)	**☆**	**☆**	**☆**	**☆**		**☆**	**☆**	**☆**	**7**
Ravikumar (2016)	**☆**	**☆**	**☆**	**☆**		**☆**	**☆**	**☆**	**7**
Selzner (2016)	**☆**		**☆**	**☆**		**☆**	**☆**	**☆**	**6**
Bral (2017)	**☆**	**☆**	**☆**	**☆**		**☆**	**☆**	**☆**	**7**
van Rijn (2017)	**☆**	**☆**	**☆**	**☆**		**☆**	**☆**	**☆**	**7**
Nasralla (2018)	**☆**	**☆**	**☆**	**☆**	**☆**	**☆**	**☆**	**☆**	**8**

## Supplementary Materials

Supplementary Materials
* Supplementary 1*. Subgroup analysis of peak AST in SNMP in animal studies.* Supplementary 2*. Subgroup analysis of animal survival in NMP in animal studies.* Supplementary 3*. Subgroup analysis of animal survival in HMP in animal studies.* Supplementary 4*. Subgroup analysis of EAD in NMP in human studies.* Supplementary 5*. Subgroup analysis of graft survival in HMP in human studies.* Supplementary 6*. Meta-analysis of hospital stay, ICU stay, peak AST, and INR in human studies.* Supplementary 7*. Publication bias in all studies using Begg's test.

## References

[1] Detelich D., Markmann J. F. (2018). The dawn of liver perfusion machines. *Current Opinion in Organ Transplantation*.

[2] Liu S., Pang Q., Zhang J., Zhai M., Liu S., Liu C. (2016). Machine perfusion versus cold storage of livers: a meta-analysis. *Frontiers of Medicine*.

[3] Jing L., Yao L., Zhao M., Peng L., Liu M. (2018). Organ preservation: from the past to the future. *Acta Pharmacologica Sinica*.

[4] Marecki H., Bozorgzadeh A., Porte R. J., Leuvenink H. G., Uygun K., Martins P. N. (2017). Liver ex situ machine perfusion preservation: A review of the methodology and results of large animal studies and clinical trials. *Liver Transplantation*.

[5] Schlegel A., Kron P., Graf R., Dutkowski P., Clavien P.-A. (2014). Warm vs. cold perfusion techniques to rescue rodent liver grafts. *Journal of Hepatology*.

[6] Jia J. J., Zhang J., Li J. H. (2015). Influence of perfusate on liver viability during hypothermic machine perfusion. *World Journal of Gastroenterology*.

[7] Bruinsma B. G., Berendsen T. A., Izamis M. L., Yeh H., Yarmush M. L., Uygun K. (2015). Supercooling preservation and transplantation of the rat liver. *Nature Protocols*.

[8] Hoyer D. P., Paul A., Luer S., Reis H., Efferz P., Minor T. (2016). End-ischemic reconditioning of liver allografts: Controlling the rewarming. *Liver Transplantation*.

[9] Nassar A., Liu Q., Farias K. (2015). Ex vivo normothermic machine perfusion is safe, simple, and reliable: Results from a large animal model. *Surgical Innovation*.

[10] Gringeri E., Bonsignore P., Bassi D. (2012). Subnormothermic machine perfusion for non-heart-beating donor liver grafts preservation in a swine model: A new strategy to increase the donor pool?. *Transplantation Proceedings*.

[11] Olthoff K. M., Kulik L., Samstein B. (2010). Validation of a current definition of early allograft dysfunction in liver transplant recipients and analysis of risk factors. *Liver Transplantation*.

[12] Chae M. S., Kim Y., Lee N. (2018). Graft regeneration and functional recovery in patients with early allograft dysfunction after living-donor liver transplantation. *Annals of Transplantation*.

[13] Kulik U., Lehner F., Klempnauer J., Borlak J. (2017). Primary non-function is frequently associated with fatty liver allografts and high mortality after re-transplantation. *Liver International*.

[14] Chen X.-B., Xu M.-Q. (2014). Primary graft dysfunction after liver transplantation. *Hepatobiliary & Pancreatic Diseases International*.

[15] Hozo S. P., Djulbegovic B., Hozo I. (2005). Estimating the mean and variance from the median, range, and the size of a sample. *BMC Medical Research Methodology*.

[16] Cota G. F., de Sousa M. R., Fereguetti T. O., Rabello A. (2013). Efficacy of Anti-Leishmania Therapy in Visceral Leishmaniasis among HIV Infected Patients: A Systematic Review with Indirect Comparison. *PLOS Neglected Tropical Diseases*.

[17] Hooijmans C. R., Rovers M. M., De Vries R. B. M., Leenaars M., Ritskes-Hoitinga M., Langendam M. W. (2014). SYRCLE's risk of bias tool for animal studies. *BMC Medical Research Methodology*.

[18] Liu Q., Nassar A., Farias K. (2014). Sanguineous normothermic machine perfusion improves hemodynamics and biliary epithelial regeneration in donation after cardiac death porcine livers. *Liver Transplantation*.

[19] Sadowsky D., Zamora R., Barclay D., Yin J., Fontes P., Vodovotz Y. (2016). Machine perfusion of porcine livers with oxygen-carrying solution results in reprogramming of dynamic inflammation networks. *Frontiers in Pharmacology*.

[20] Li P., Liu Y.-F., Yang L. (2015). Advantages of dual hypothermic oxygenated machine perfusion over simple cold storage in the preservation of liver from porcine donors after cardiac death. *Clinical Transplantation*.

[21] Compagnon P., Levesque E., Hentati H. (2017). An oxygenated and transportable machine perfusion system fully rescues liver grafts exposed to lethal ischemic damage in a pig model of DCD liver transplantation. *Transplantation*.

[22] Schön M. R., Kollmar O., Wolf S. (2001). Liver transplantation after organ preservation with normothermic extracorporeal perfusion. *Annals of Surgery*.

[23] Brockmann J., Reddy S., Coussios C. (2009). Normothermic perfusion: a new paradigm for organ preservation. *Annals of Surgery*.

[24] Goldaracena N., Echeverri J., Spetzler V. N. (2016). Anti-inflammatory signaling during ex vivo liver perfusion improves the preservation of pig liver grafts before transplantation. *Liver Transplantation*.

[25] Dutkowski P., Polak W. G., Muiesan P. (2015). First comparison of hypothermic oxygenated perfusion versus static cold storage of human donation after cardiac death liver transplants: An international-matched case analysis. *Annals of Surgery*.

[26] Iwamoto H., Matsuno N., Narumi Y. (2000). Beneficial effect of machine perfusion preservation on liver transplantation from non-heart-beating donors. *Transplantation Proceedings*.

[27] Fondevila C., Hessheimer A. J., Maathuis M.-H. J. (2011). Superior preservation of DCD livers with continuous normothermic perfusion. *Annals of Surgery*.

[28] Monbaliu D., Heedfeld V., Liu Q. (2011). Hypothermic machine perfusion of the liver: Is it more complex than for the kidney?. *Transplantation Proceedings*.

[29] Fondevila C., Hessheimer A. J., Maathuis M.-H. J. (2012). Hypothermic oxygenated machine perfusion in porcine donation after circulatory determination of death liver transplant. *Transplantation*.

[30] Knaak J. M., Spetzler V. N., Goldaracena N. (2014). Subnormothermic ex vivo liver perfusion reduces endothelial cell and bile duct injury after donation after cardiac death pig liver transplantation. *Liver Transplantation*.

[31] Fontes P., Lopez R., Van Der Plaats A. (2015). Liver preservation with machine perfusion and a newly developed cell-free oxygen carrier solution under subnormothermic conditions. *American Journal of Transplantation*.

[32] Spetzler V. N., Goldaracena N., Echiverri J. (2016). Subnormothermic ex vivo liver perfusion is a safe alternative to cold static storage for preserving standard criteria grafts. *Liver Transplantation*.

[33] Zhang Z.-B., Gao W., Shi Y. (2016). Protective role of normothermic machine perfusion during reduced-size liver transplantation in pigs. *Liver Transplantation*.

[34] Guarrera J. V., Henry S. D., Samstein B. (2010). Hypothermic machine preservation in human liver transplantation: The first clinical series. *American Journal of Transplantation*.

[35] Guarrera J. V., Henry S. D., Samstein B. (2015). Hypothermic machine preservation facilitates successful transplantation of "orphan" extended criteria donor livers. *American Journal of Transplantation*.

[36] Ravikumar R., Jassem W., Mergental H. (2016). Liver transplantation after ex vivo normothermic machine preservation: A phase 1 (first-in-man) clinical trial. *American Journal of Transplantation*.

[37] Selzner M., Goldaracena N., Echeverri J. (2016). Normothermic ex vivo liver perfusion using steen solution as perfusate for human liver transplantation: First north american results. *Liver Transplantation*.

[38] Bral M., Gala-Lopez B., Bigam D. (2017). Preliminary single-center canadian experience of human normothermic ex vivo liver perfusion: Results of a clinical trial. *American Journal of Transplantation*.

[39] van Rijn R., Karimian N., Matton A. P. M. (2017). Dual hypothermic oxygenated machine perfusion in liver transplants donated after circulatory death. *British Journal of Surgery*.

[40] Nasralla D., Coussios C. C., Mergental H. (2018). A randomized trial of normothermic preservation in liver transplantation. *Nature*.

[41] Bian S., Zhu Z., Sun L. (2018). Normothermic machine perfusion versus cold storage of liver in pig model: A meta-analysis. *Annals of Transplantation*.

[42] Ceresa C. D. L., Nasralla D., Coussios C. C., Friend P. J. (2018). The case for normothermic machine perfusion in liver transplantation. *Liver Transplantation*.

[43] Okamura Y., Hata K., Tanaka H. (2017). Impact of subnormothermic machine perfusion preservation in severely steatotic rat livers: A detailed assessment in an isolated setting. *American Journal of Transplantation*.

[44] Russo L., Gracia-Sancho J., García-Calderó H. (2012). Addition of simvastatin to cold storage solution prevents endothelial dysfunction in explanted rat livers. *Hepatology*.

[45] Schlegel A., Kron P., Dutkowski P. (2016). Hypothermic machine perfusion in liver transplantation. *Current Opinion in Organ Transplantation*.

[46] Schlegel A., Graf R., Clavien P.-A., Dutkowski P. (2013). Hypothermic oxygenated perfusion (HOPE) protects from biliary injury in a rodent model of DCD liver transplantation. *Journal of Hepatology*.

