# Supplementary material for: Optimizing Livers for Transplantation Using Machine Perfusion versus Cold Storage in Large Animal Studies and Human Studies: A Systematic Review and Meta-Analysis
Source: Biomed Res Int. 2018 Sep 5;2018:9180757. doi: 10.1155/2018/9180757 (PMC6145150; doi:10.1155/2018/9180757)
Supplement: Supplementary Materials — Supplementary 1. Subgroup analysis of peak AST in SNMP in animal studies. Supplementary 2. Subgroup analysis of animal survival in NMP in animal studies. Supplementary 3. Subgroup analysis of animal survival in HMP in animal studies. Supplementary 4. Subgroup analysis of EAD in NMP in human studies. Supplementary 5. Subgroup analysis of graft survival in HMP in human studies. Supplementary 6. Meta-analysis of hospital stay, ICU stay, peak AST, and INR in human studies. Supplementary 7. Publication bias in all studies using Begg's test. [file 9180757.f1.pdf]

Supplementary materials

Supplementary 1. Subgroup analysis of peak AST in SNMP in animal studies

Subgroup	No. of studies	Subtotal SMD(95%CI)	I^2 value	P value
**WIT**				
NR	2	-0.61(-1.40,0.18)	0.0%	0.133
30-60min	2	-0.68(-3.28,1.92)	86.3%	0.610
**Donor type**				
HBD	1	-0.46(-1.72,0.80)	--	0.474
DCD	3	-0.67(-2.04,0.70)	72.9%	0.339

Supplementary 2. Subgroup analysis of animal survival in NMP in animal studies

Subgroup	No. of studies	Subtotal RR(95%CI)	I^2 value	P value
**WIT**				
NR	1	1.000(0.538,1.859)	--	1.000
0min	2	1.581(0.450,5.555)	71.6%	0.475
0-30min	1	1.182(0.763,1.831)	--	0.455
30-60min	1	7.857(0.551,112.089)	--	0.128
**Donor type**				
HBD	3	1.225(0.670,2.240)	47.2%	0.510
DCD	2	2.539(0.136,47.501)	79.6%	0.533

Supplementary 3. Subgroup analysis of animal survival in HMP in animal studies

Subgroup	No. of studies	Subtotal RR(95%CI)	I^2 value	P value
**WIT**				
NR	2	0.388(0.128,1.178)	0.0%	0.095
0-30min	1	10.50(0.698,157.908)	--	0.086

Supplementary 4. Subgroup analysis of EAD in NMP in human studies

Subgroup	No. of studies	Subtotal RR(95%CI)	I^2 value	P value
**Donor type**				
DCD	1	0.677(0.203,2.194)	--	0.505
DCD/DBD	2	0.788(0.143,4.354)	90.9%	0.784

Supplementary 5. Subgroup analysis of graft survival in HMP in human studies

Subgroup	No. of studies	Subtotal RR(95%CI)	I^2 value	P value
**WIT**				
NR	2	1.003(0.856,1.176)	0.0%	0.968
0-30min	1	1.743(1.157,2.265)	--	0.008
30-60min	2	1.111(0.792,1.558)	86.3%	0.542
**Donor type**				
DCD	2	1.288 (0.716,2.314)	84.8%	0.398
ECD	1	1.008(0.786,1.292)	--	0.949

DCD/DBD	2	1.111(0.792,1.558)	86.3%	0.542

Supplementary 6. Meta- analysis of hospital stay, ICU stay, peak AST and INR in human studies

**Subgroup**	**No. of studies**	**Subtotal SMD(95%CI)**	**I^2 value**	**P value**
**Hospital stay**				
NMP	3	0.338 (-0.286 to 0.963)	60.9%	0.288
HMP	5	-0.088 (-0.662 to 0.485)	80.6%	0.762
**ICU stay**				
NMP	3	0.563(-0.330 to 1.457)	80.0%	0.217
HMP	3	0.307 (-0.004 to 0.619)	0.0%	0.053
**Peak AST**				
NMP	3	-4.616(-10.364 to 1.133)	99.3%	0.116
HMP	3	-1.235(-3.333 to 0.863)	97.0%	0.249
**INR**				
NMP	4	0.083(-0.133 to 0.299)	0.0%	0.453
HMP	3	-0.594(-2.683 to 1.495)	97.3%	0.577

Supplementary 7. Publication bias in all studies using Begg's test

**Subgroup**	**No. of studies**	**P-Q**	**Std. Dev. of Score**	**P value**
**Peak AST(animal)**				
NMP	2	-1	1.00	0.317
SNMP	4	0	2.94	1.000
HMP	2	1	1.00	0.317
**HA**				
NMP	2	-1	1.00	0.317
HMP	2	-1	1.00	0.317
**Animal survival**				
NMP	5	3	4.08	0.463
SNMP	4	4	2.94	0.174
HMP	3	3	1.91	0.117
**Biliary complication**				
NMP	4	2	2.94	0.497
HMP	5	-2	4.08	0.624
**EAD**				
NMP	3	1	1.91	0.602
HMP	4	0	2.94	1.000
**PNF**				
NMP	1	--	--	--
HMP	2	-1	1.00	0.317
**Graft survival**				
NMP	2	1	1.00	0.317
HMP	5	8	4.08	0.050
**Hospital stay**				
NMP	3	1	1.91	0.602

HMP	5	-4	4.08	0.327
**ICU stay**				
NMP	3	3	1.91	0.117
HMP	3	3	1.91	0.117
**Peak AST(human)**				
NMP	3	-3	1.91	0.117
HMP	3	-1	1.91	0.602
**INR**				
NMP	4	4	2.94	0.174
HMP	3	-1	1.91	0.602
